# Water‐soluble Sr_4_Al_2_O_7_ and its possible applications in medicine research

**DOI:** 10.1002/ctm2.1641

**Published:** 2024-04-01

**Authors:** Nan Liu, Liang Si, Wen‐Li Yang

**Affiliations:** ^1^ School of Physics Northwest University Xi'an China; ^2^ Shaanxi Key Laboratory for Theoretical Physics Frontiers Xi'an China; ^3^ Peng Huanwu Center for Fundamental Theory Xi'an China

**Keywords:** functional oxide, luminescent material, water‐soluble oxide

Recently, a new water‐soluble sacrificial‐layer oxide: Sr_4_Al_2_O_7_, has been identified.^1^, ^2^ Sr_4_Al_2_O_7_ has better structural and thermodynamic stability, and it also possesses a pseudo‐cubic lattice constant closer to perovskite ABO_3_ systems. Additionally, in comparison to Sr_3_Al_2_O_6_,^3^ Sr_4_Al_2_O_7_ exhibits a faster water‐soluble rate and excellent structural elasticity. Its excellent structural elasticity facilitates the transfer of substrate lattice to target oxide membranes. Theoretical simulations have further revealed that a lower crystal symmetry of Sr_4_Al_2_O_7_ and higher bonding energy at the interface between Sr_4_Al_2_O_7_ and ABO_3_ heterojunctions, compared to Sr_3_Al_2_O_6_, thereby minimizing the increase in internal energy under strain and promoting the formation of complete atomic‐level interfaces and enabling the synthesis of high‐quality free‐standing oxide membranes.

## 1 USING SR_4_AL_2_O_7_ TO ENHANCE THE QUALITY OF FREE‐STANDING OXIDE MEMBRANES

**FIGURE 1:**
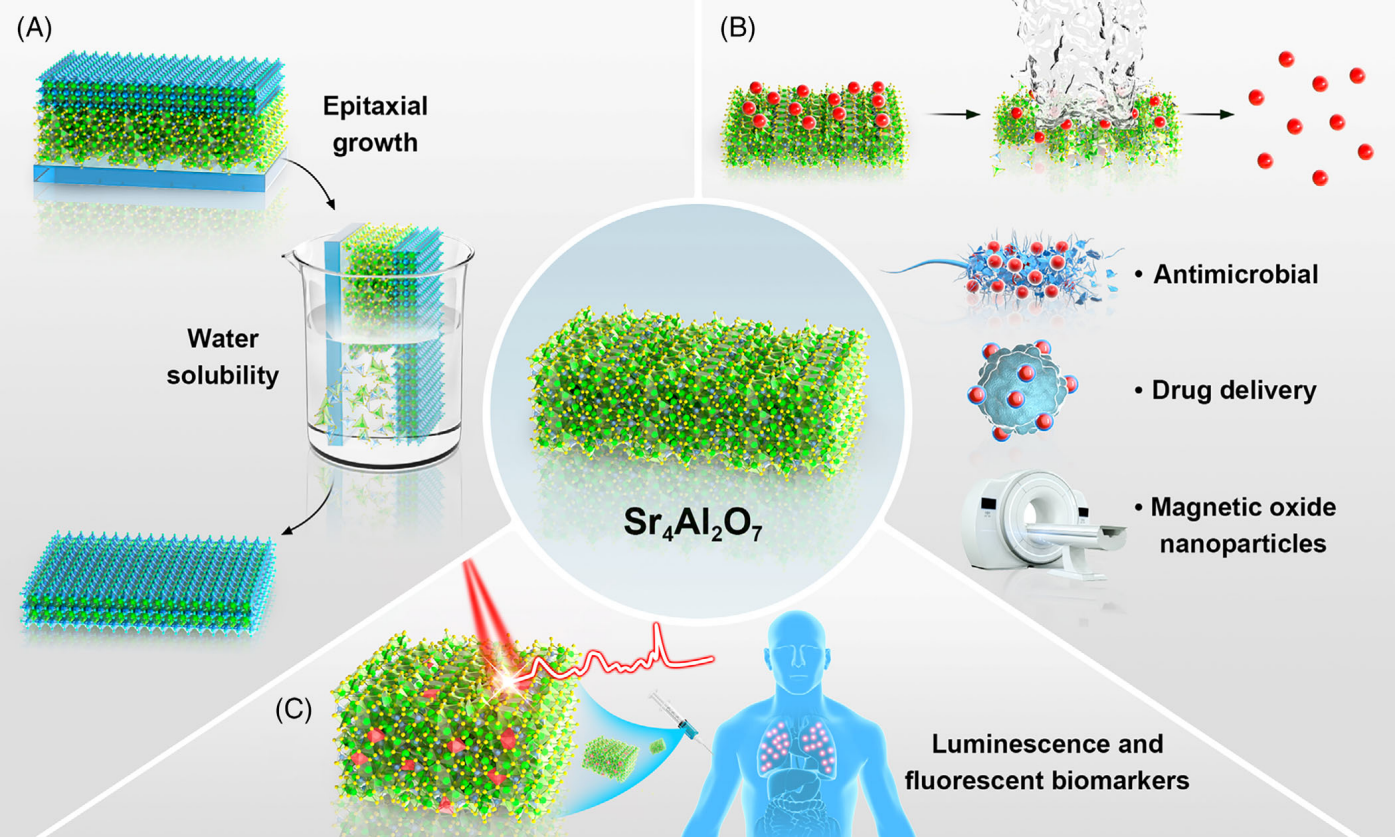
(A) Synthesis of free‐standing oxide membranes using Sr_4_Al_2_O_7_ as sacrificial layer; (B) utilizing Sr_4_Al_2_O_7_ for medical nanoparticle synthesis, and potential applications; (C) potential medical applications of rare‐earth elements doped Sr_4_Al_2_O_7_.

Utilizing Sr_4_Al_2_O_7_ as a sacrificial layer for the synthesis of free‐standing oxide membranes has shown promising results across various oxide materials (Figure 1A). These membranes, including nickelate catalyst LaNiO_3_, ferromagnetic (La,Ca)MnO_3_, dielectric substrate SrTiO_3_, electrode material SrRuO_3_, and photocatalyst SrSnO_3_, exhibit exceptional crystal quality and integrity, along with larger sample sizes to the millimetre level. Moreover, they exhibit physical and chemical properties comparable to corresponding epitaxial thin films. These advancements suggest that the successful synthesis of Sr_4_Al_2_O_7_ will not only significantly enhance the rate and quality of the synthesis of oxide membranes but also offer potential benefits in the fabrication of (1) oxide nanoparticles, as well as (2) rare‐earth elements doped Sr_4_Al_2_O_7_, both of these systems are anticipated to play significant roles in medicine applications (Figure 1B, C).

## 2 UTILIZING SR_4_AL_2_O_7_ FOR MEDICAL NANOPARTICLE SYNTHESIS

In recent years, the field of medicine research has shown a surge in interest in the application of oxide nanoparticles and membranes across a wide range of healthcare applications (Figure 1B). From antimicrobial coatings to drug delivery systems (Figure 1B), the unique properties of nano‐size oxides offer the potential to revolutionize various aspects of healthcare: (1) Magnetic oxide nanoparticles (such as Fe_3_O_4_) play a crucial role in targeted imaging and contrast enhancement in medical imaging techniques such as magnetic resonance imaging and computed tomography scans^4^ (Figure 1C). Additionally, the development of oxide‐based biosensors (such as MgO) enables the detection of biomarkers and pathogens, facilitating early disease diagnosis and monitoring. (2) Nanoparticles such as titanium dioxide (TiO_2_) offer tailored properties suitable for drug delivery applications, due to their high surface area, tunable pore sizes, biocompatibility and controlled release capabilities.^5^ Additionally, TiO_2_ is utilized in photodynamic therapy for cancer treatment, leveraging its photophysical properties for localized tumour ablation. (3) Antimicrobial oxides, including zinc oxide and cuprates nanoparticles, possess broad‐spectrum antimicrobial activity against bacteria, viruses, and fungi, making them viable candidates for developing antimicrobial coatings for medical devices and surfaces.^6^

The discovery and synthesis of Sr_4_Al_2_O_7_ as a sacrificial layer promise to revolutionize the production of free‐standing low‐dimensional systems comprising various perovskite oxides, such as their nanoparticles and membranes, enhancing both the rate and quality of their synthesis. This breakthrough, coupled with the diverse physical and chemical properties of perovskite oxides, will expand the select range of materials available for the aforementioned applications. For instance, room‐temperature ferromagnetic nanoparticles of (La,Ca)MnO_3_ could potentially replace Fe_2_O_3_, while nanoparticles of insulating SrTiO_3_ might offer an alternative to TiO_2_. These functional oxide nanoparticles hold significant implications for medical applications, providing a broader spectrum of choices to cater to specific needs and requirements.

## 3 POTENTIAL MEDICAL APPLICATIONS OF RARE‐EARTH ELEMENTS DOPED SR_4_AL_2_O_7_

Rare‐earth elements doped Sr_4_Al_2_O_7_ (also Sr_3_Al_2_O_6_) hold significant promise for various medical applications owing to their unique magnetic and luminescent properties (Figure 1C). Some key potential applications include: (1) Rare‐earth doped insulators function as fluorescent biomarkers for cellular and molecular imaging applications.^7^ The luminescent properties of rare‐earth ions, such as europium (Eu) and terbium (Tb), facilitate multiplexed imaging, deep‐tissue imaging, and single‐molecule tracking of biological processes and biomolecules in living cells and tissues. (2) Rare‐earth doped insulators act as carriers for drug delivery systems in medicine.^8^ Utilizing the luminescent properties of rare‐earth ions enables real‐time monitoring of drug release and distribution in biological tissues and organs. (3) Rare‐earth doped insulators find applications in biosensors for detecting and quantifying biomolecules, pathogens, and environmental pollutants.^9^ Functionalization of rare‐earth‐doped nanoparticles with recognition elements, such as antibodies or aptamers, enables selective binding to specific biomarkers or pathogens for biosensing applications. (4) Rare‐earth doped insulators serve as photosensitizers in photodynamic therapy for cancer treatment.^10^ The luminescent properties of rare‐earth ions enable the generation of reactive oxygen species upon light irradiation, leading to the selective destruction of cancer cells while minimizing damage to healthy tissues.

Given its robust thermal and structural stability, doped Sr_4_Al_2_O_7_ holds promise for achieving stable crystal structures and spatial arrangements of doped rare‐earth elements through chemical doping on the Sr‐site. With the participation of rare‐earth element ions, properties such as luminescence and magnetism are anticipated to manifest. Consequently, this development will introduce novel candidates for rare earth element‐doped oxide materials with potential applications in medicine.

## 4 FURTHER INVESTIGATION

The successful identification and synthesis of Sr_4_Al_2_O_7_ as an effective sacrificial layer is poised to unlock a new realm of functional oxide materials research. With its stable insulator and sacrificial‐layer nature, Sr_4_Al_2_O_7_ holds significant promise in the synthesis of low‐dimensional oxide compounds, such as membranes and nanoparticles, thereby possibly contributing to disease diagnosis, treatment, and biomedical research. Furthermore, the potential applications of rare‐earth element doped Sr_4_Al_2_O_7_ warrant exploration, including its utilization in phosphors, bioimaging, fluorescent biomarkers, and drug delivery systems. Further research endeavours aim to unravel the full spectrum of applications and optimize the performance of Sr_4_Al_2_O_7_‐based oxides for medical applications across diverse fields.

## AUTHOR CONTRIBUTIONS

All authors have contributed to writing the paper, creating visualizations, and have read and approved the final manuscript.

## ETHICS STATEMENT

Not Applicable.
